# Effects of Eye Drops Containing Hyaluronic Acid-Nimesulide Conjugates in a Benzalkonium Chloride-Induced Experimental Dry Eye Rabbit Model

**DOI:** 10.3390/pharmaceutics13091366

**Published:** 2021-08-30

**Authors:** Tzu-Yang Chen, Ching-Li Tseng, Chih-An Lin, Hua-Yang Lin, Parthiban Venkatesan, Ping-Shan Lai

**Affiliations:** 1 Department of Chemistry, National Chung Hsing University, Taichung 40227, Taiwan; cypanjimmy@gmail.com (T.-Y.C.); venkatesanwala@gmail.com (P.V.); 2 Graduate Institute of Biomedical Materials & Tissue Engineering, College of Biomedical Engineering, Taipei Medical University, Taipei 11031, Taiwan; chingli@tmu.edu.tw; 3 Ph.D. Program of Tissue Engineering and Regenerative Medicine, National Chung Hsing University, Taichung 40227, Taiwan; fire0082@gmail.com; 4 Preclinical Development Research Department, Holy Stone Healthcare Co., Ltd., Taipei 11493, Taiwan; louislin@hshc.com.tw

**Keywords:** ocular, topical administration, COX-2 inhibitor, anti-inflammatory, solvent-free

## Abstract

Dry eye syndrome (DES) is a common ocular disease worldwide. Currently, anti-inflammatory agents and immunosuppressive drugs, such as cyclosporine A, have been widely used to treat this chronic condition. However, the multifactorial etiology of DES, poor tolerance, low bioavailability, and prolonged treatment to response time have limited their usage. In this study, nimesulide, a cyclooxygenase (COX)-2 selective inhibitor, was conjugated with hyaluronic acid (HA), and the HA-nimesulide conjugates were expected to increase the solubility and biocompatibility for alleviating the DES in the benzalkonium chloride (BAC)-induced goblet cell-loss dry eye model. The therapeutic efficacy of HA-nimesulide was assessed using fluorescein staining, goblet cell density by conjunctival impression cytology, and histology and immunohistochemistry of corneal tissues. Compared to commercial artificial tears and Restasis^®^, the HA-nimesulide conjugates could promote goblet cell recovery and enhance the regeneration of the corneal epithelium. Importantly, immunofluorescent staining studies demonstrated that the HA-nimesulide conjugates could decrease the number of infiltrating CD11b-positive cells after two weeks of topical application. In the anti-inflammatory test, the HA-nimesulide conjugates could inhibit the production of pro-inflammatory cytokines and prostaglandin E_2_ (PGE2) in the lipopolysaccharide (LPS)-stimulated Raw 264.7 cell model. In conclusion, we demonstrated that HA-nimesulide conjugates had anti-inflammatory activity, and promoted goblet cell recovery and corneal epithelium regeneration when used as topical eye drops; accordingly, the HA-nimesulide conjugates could potentially be effective for the treatment of DES.

## 1. Introduction

Dry eye syndrome (DES) is a very common ocular disease worldwide, the prevalence of which ranges from 5% to 50% and is rising with the increasingly aging population. It is estimated that 7 to 10 million people have spent $320 US dollars per person per year on artificial tears alone in the United States [1,2]. DES is also known as keratoconjunctivitis sicca (KCS), and it is a chronic tear and ocular surface disorder of multiple etiologies. The symptoms of DES can be severely disabling, which may include discomfort, stinging, burning, sandy or gritty sensation, episodic blurred vision, excessive tearing, and redness [3]. Tear hyperosmolarity and tear film instability from high evaporation and low lacrimal flow are considered as the causes of DES [4], which leads to corneal and conjunctival epithelial damage from an array of inflammatory mediators and related cytokines, and in turn contributes to the unstable tear film, forming an unremitting vicious cycle [5].

Currently, anti-inflammatory agents such as cyclosporine A and corticosteroids have been approved to treat DES, but not without limitations. For example, the risk of systemic side effects, glaucoma, and cataracts associated with long-term corticosteroid treatment [6] and the intolerable side effects such as pain, burning, and irritation linked to cyclosporine A. Prabhasawat et al. reported that 23% of all patients discontinued cyclosporine A within the first 3 months because of side effects [7], and these side effects might result from the castor oil-based formulation in the Restasis^®^ ophthalmic emulsion [8]. In addition to the side effects, the treatment efficacy may be hindered by the ocular surface tear film barriers such as a high turnover rate and mucus layer [9,10]. These two characteristics decrease drug concentration and prevent contact between the drug molecules and the ocular surface. Therefore, a new treatment strategy to alleviate the side effects or reduce the influence from the physical barriers is necessary for topical administration.

Several topical ophthalmic nonsteroidal anti-inflammatory drugs (NSAIDs), such as diclofenac ophthalmic solution, flurbiprofen ophthalmic solution, and ketorolac ophthalmic solution, are generally used in cataract surgery, refractive surgery, and seasonal allergic conjunctivitis [11,12]. Nimesulide is an NSAID and selective inhibitor for cyclooxygenase (COX)-2, inhibiting the production of prostaglandins. In ophthalmology, oral administration of nimesulide can increase the therapeutic efficacy of latanoprost when treating glaucoma with increased intraocular pressure [13]. El-Shazly et al. reported, in an experimental dry eye model, the beneficial effects of topical nimesulide and ketorolac treatment with respect to Schirmer test values, tear break-up time, fluorescein corneal staining, inflammation, and regeneration, and suggested the use of nimesulide to avoid the local and systemic side effects seen with prolonged and repeated administration of non-selective COX inhibitors, however, the bioavailability of nimesulide was low as a result of poor aqueous solubility [14].

Hyaluronic acid (HA) is an anionic polysaccharide made of disaccharide units and is naturally abundant in the human body, such as in skin, joints, and vitreous humor. Therefore, HA has several biomedical applications, such as in cosmetics [15], drug delivery [16], and a common component of commercial artificial tears [17,18,19], due to its hydrophilic nature, good biocompatibility, nonirritating property, and mucoadhesive ability to facilitate the interaction with the tear mucin layer. The mucoadhesive property of HA could prolong the ocular residence time [20], and HA could target cell surface receptors such as the cluster determinant 44 (CD44), which is expressed in rabbit and human cornea [21]. HA may be used as a drug carrier in drug delivery systems; most frequently, the direct conjugation between HA and drugs is employed to enhance the solubility and hence the bioavailability of hydrophobic drugs [22,23]. Furthermore, these HA conjugates have been used as carriers for sustained release in dry eye treatment [24].

**Figure 1:**
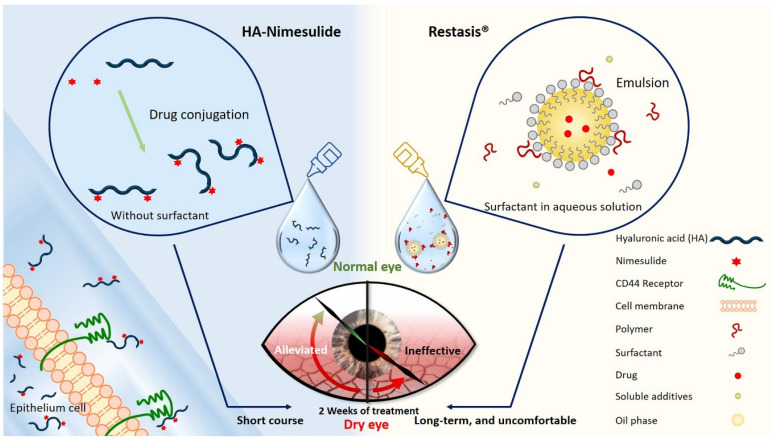
Schematic diagram illustrating the novel HA-nimesulide conjugate system and the standard Restasis^®^ to treat dry eye syndrome. The components of each formulation are depicted. HA-nimesulide has the advantage of being surfactant-free, which is thought to cause irritating eye symptoms, and improves the bioavailability of nimesulide; furthermore, CD44 receptors on the epithelial cells are targeted for greater drug retention time.

In this study, we developed a surfactant-free and aqueous-based HA-nimesulide conjugate as eye drops for ocular applications (Figure 1), and this formulation was expected to improve patient compliance through a decrease of eye discomfort. The therapeutic effects of the HA-nimesulide conjugate were evaluated in a rabbit model of benzalkonium chloride (BAC)-induced goblet cell loss, and the possible anti-inflammatory mechanisms of the HA-nimesulide conjugate in Raw 264.7 cells were studied in vitro.

## 2. Materials and Methods

### 2.1. Materials

HA (HA with high molecular weight, 360 kDa (HAH), and HA with low molecular weight, 36 kDa (HAL); Freda Biopharm, Tsingtao, China) was provided by HolyStone HealthCare (Taipei, Taiwan). Nimesulide, 1-ethyl-3-(3-dimethylaminopropyl)carbodiimide (EDC) hydrochloride, adipic acid dihydrazide (ADH), N-hydroxysuccinimide (NHS), ethyl (hydroxyimino)cyanoacetate (Oxyma), dimethyl sulfoxide (DMSO), fluorescein sodium salt, Griess reagent (G4410), and lipopolysaccharide (LPS, *Escherichia coli.* O55:B5, L2880) were purchased from Sigma-Aldrich (St. Louis, MO, USA). BAC was purchased from Acros Organics (Geel, Belgium). Zoletil 50 was purchased from Virbac (Carros, France), and Rompun 20 was purchased from Bayer Korea Ltd. (Seoul, Korea). Alcaine 0.5% was purchased from Alcon (Fort Worth, TX, USA). Optive Fusion^®^ and Restasis^®^ were purchased from Allergan (Mayo, Ireland). Rat IgG anti-rabbit CD11b (M1/70; ab8878) was purchased from Abcam (Cambridge, UK). Alexa Fluor^®^ 488 donkey anti-rat IgG (H + L) (A21208), 3-(4,5-dimethylthiazolyl)-2-2,5-diphenyltetrazolium bromide (MTT), interleukin-6 ELISA kit (88-7064-88), and tumor necrosis factor alpha ELISA kit (88-7324-88) were purchased from Invitrogen (Waltham, MA, USA). Hoechst 33342 was purchased from BD (Franklin Lakes, NJ, USA). Dulbecco’s Modified Eagle Medium (DMEM, 12100-046, Gibco), fetal bovine serum (FBS, 10437-028, Gibco), penicillin (PSN, 15640-055, Gibco), and sodium pyruvate (11360-070, Gibco) were purchased from Thermo Fisher Scientific (Waltham, MA, USA). Prostaglandin E_2_ ELISA Kit (KA0326) was purchased from Abnova (Taipei, Taiwan). All other reagents were of guaranteed grade.

### 2.2. Synthesis of HA-Nimesulide Conjugates

HA-conjugated nimesulide was synthesized as described in our previous report [23]. Briefly, the nitro group of the nimesulide functional group was first hydrogenated to an amine group under atmospheric pressure at room temperature in the presence of Pd/C for 24 h. The synthetic steps of the low molecular weight HA-nimesulide conjugate (H1) are shown as follows: (1) 36 kDa HA (1 g, 2.488 mmol) was dissolved in 100 mL of deionized water and 140 mL of DMSO, (2) nimesulide-NH_2_ (31.2 mg, 0.112 mmol) dissolved in 10 mL of DMSO, and Oxyma (354.0 mg, 2.493 mmol) dissolved in 10 mL of DMSO, were added, and (3) a total of 810 μL of DIC dissolved in 10 mL of DMSO was added and reacted at room temperature for 24 h. The solution was transferred to a dialysis tubing (Molecular weight cut off, MWCO 3500) and dialyzed against 0.3 M NaCl solution and water for 4 days. The resulting solution was lyophilized and stored at 4 °C for further studies. The molecular weight of HA was changed from 36 to 360 kDa for the synthesis of the high molecular weight HA-nimesulide conjugate (H2), and the weight of nimesulide was increased from 31.2 mg (0.112 mmol) to 89.9 mg (0.323 mmol) for coupling reactions in the synthesis of the H3 HA-nimesulide conjugate. The grafting ratio of HA-nimesulide conjugates was defined as the number of nimesulide molecules per HA molecule obtained by ^1^H NMR spectroscopy (Vnmr-400 MHz, Agilent) in our previous report [23].

### 2.3. Characterization of HA-Nimesulide Conjugate Solutions

In the commercial artificial tears product with HA addition (0.1%), the HA concentration was adjusted to 0.1% by normal saline. The osmolarity of the HA-nimesulide conjugate solution was determined using a vapor pressure osmometer (Model 5600, Wescor EliTechGroup, Stanwood, WA, USA).

### 2.4. Animals

All of the procedures were approved by the Institutional Animal Care and Use Committee (IACUC) of National Chung Hsing University (IACUC approval no. 106-065^R^). Fourteen male New Zealand white rabbits (purchased from Livestock Research Institute, Council of Agriculture, Executive Yuan, Taiwan) weighing between 2.0 and 2.5 kg were used for the study. The rabbits were randomly divided into 6 groups: a normal control and two commercial eye drop groups: Optive Fusion^®^ and Restasis^®^. The experimental groups include three different HA-nimesulide conjugates (H1, H2, H3). All rabbits were housed at a room temperature of 23 ± 2 °C with relative humidity 75% ± 10% and alternating 12-hour light–dark cycles (8 a.m. to 8 p.m.), and all rabbits were provided food and water ad libitum.

The induction of DES was performed as previously described with slight modifications [25]. Both eyes were treated thrice daily (9 a.m., 1 p.m., and 5 p.m.) with a topical BAC solution (20 μL of 0.1%) for 4 weeks. The treatments began on day 29, with 20 μL twice daily (9 a.m., 5 p.m.) of topical Optive Fusion^®^ artificial tears, 0.1% HAL-nimesulide conjugates with a 4% grafting ratio (H1) in 0.9% normal saline, 0.1% HAH-nimesulide conjugates with a 4% grafting ratio (H2) in 0.9% normal saline, 1.0% HAL-nimesulide conjugates with a 12.5% grafting ratio (H3) in 0.9% normal saline, and Restasis^®^. No treatment was administered to the normal group. Except for the commercial artificial tears and Restasis^®^, all solutions were preservative-free and sterilized via filtration with a 0.22 μm nylon filter. The Schirmer II test, fluorescein staining, and conjunctival impression cytology were performed on days 0, 28, and 42. Rabbits were sacrificed by general anesthesia and CO_2_ euthanasia after the last measurement, and the corneal tissue was collected for further analysis. All of the procedures were performed under general anesthesia via an intramuscular injection of Rompun 20 and Zoletil 50 (2:1 ratio, 1 mL/kg).

### 2.5. Measurement of Aqueous Tear Production

Tear production was measured using Schirmer test strips on days 0, 28, and 42. After the topical application of Alcaine (0.5% proparacaine), the lower eyelid was pulled down, and a Schirmer paper strip was placed on the palpebral conjunctiva near the junction of the middle and outer thirds of the lower eyelid. After 5 min, the wetted length (mm) of the paper strips was recorded.

### 2.6. Fluorescein Staining on the Ocular Surface

Corneal fluorescein staining was performed after 2 μL of 1% fluorescein sodium was dropped into the conjunctival sac for 2 min. The ocular surface was examined under a slit lamp microscope with a cobalt blue filter (BI900, Haag-Streit, Köniz BE, Schweitzer). The images were collected by a digital camera.

### 2.7. Conjunctival Impression Cytology

Conjunctival impression cytology was performed after fluorescein staining. Samples were collected from the surface of the palpebral conjunctiva (near the junction of the middle and outer third of the lower eyelid) 2 mm lateral to the corneal limbus before and after BAC induction, and again after treatment. To obtain the sample, the ocular surface was additionally anesthetized with topical 0.5% Alcaine, and a 3.5 mm disc-shaped nitrocellulose filter paper with a 0.45 μm pore size folded in half was placed. The edge of the membranes was grasped by forceps, and light pressure was applied by the tips of the forceps. The paper was gently lifted and immediately fixed in fresh 4% paraformaldehyde for at least 30 min. Periodic acid-Schiff and hematoxylin reagents were used to stain the nitrocellulose paper, as previously described [26]. After staining, the morphology of goblet cells was collected under a microscope (Leica Microsystems Nussloch GmbH, Nussloch, Germany) with 20× and 40× objectives, and the goblet cell density was counted by ImageJ. Three different sections of each specimen were selected randomly for counting, and an average was calculated (cells/high-power (HP) visual field with 200×).

### 2.8. Histological Staining of the Cornea

The corneas from sacrificed animals were fixed in 4% paraformaldehyde for 24 h. Briefly, the specimens were dehydrated, embedded in paraffin, cross-sectioned, and stained with hematoxylin and eosin. Each corneal section morphology was examined by light microscopy.

### 2.9. Immunofluorescent Staining

The corneal sections were immersed in xylene 3 times for 3 min for deparaffinization and then immersed in a gradient concentration of alcohol for rehydration. The sections were washed in deionized water and immersed in pH 6.0 and 10 mM citrate buffer at 90 °C for 30 min for antigen retrieval. After blocking in 3% H_2_O_2_ and 5% bovine serum albumin (BSA), immunofluorescence staining was performed with the following primary antibody: rat IgG anti-rabbit CD11b (1:200). The secondary antibodies included the following: Alexa Fluor^®^ 488 donkey anti-rat IgG (H + L) (1:500). After 3 washes with deionized water, the sections were further incubated with Hoechst 33342 (1:2000) for 5 min. The fluorescence signal was detected under a fluorescence microscope (FV1200 confocal microscopy system, Olympus, Tokyo, Japan).

### 2.10. Cell Culture Condition

The Raw 264.7 cell line was purchased from Bioresource Collection and Research Centre (Hsinchu, Taiwan). Raw 264.7 cells were cultured in DMEM with 10% of FBS, 1% of PSN, and 1% of sodium pyruvate, and incubated at 37 °C and 5% CO_2_.

### 2.11. Cell Viability Assay

The viability of Raw 264.7 cells was determined using the MTT reagent. The cells at a density of 1 × 10^4^ cells/well were seeded in 96-well plates. After a 24 hour incubation, various concentrations of nimesulide or equivalent of nimesulide in HA conjugates (400 μM to 25 μM) were added to the cells and incubated for an additional 24 h. The MTT solution was added to each well and incubated for 4 h at 37 °C. After removing the MTT solution, DMSO was added to each well to dissolve the formazan. The absorbance was measured using a scanning multi-well plate reader at 570 nm (SpectraMax^®^ M2e, Molecular Devices LLC, San Jose, CA, USA).

### 2.12. Nitrite Concentration Determination

The nitrite concentration in the cell culture medium was determined using the Griess reagent according to the manufacturer’s instructions. Briefly, the cells at a density of 2.5 × 10^5^ cells/well were seeded in 6-well plates and incubated for 24 h. The cells were stimulated with or without 1 μg/mL of LPS for 30 min and then incubated with 50 μM of nimesulide or the equivalent of nimesulide in HA conjugates for 24 h [27]. After incubation, culture medium was collected and centrifuged (3000× *g*, 10 min), and 100 μL of supernatant was mixed with an equal volume of Griess reagent for 15 min at room temperature. Fresh culture medium was used as a blank in all experiments. The absorbance was measured using a scanning multi-well plate reader at 540 nm, and the amount of nitrite in the culture medium was estimated via a standard curve of sodium nitrite.

### 2.13. Cytokines and PGE2 Concentration Determination

The interleukin-6 (IL-6), tumor necrosis factor alpha (TNF-α), and prostaglandin E_2_ (PGE2) metabolites in the cell culture medium were measured using an enzyme immunoassay kit according to the manufacturer’s instructions. The cells at a density of 2.5 × 10^5^ cells/well were seeded in 6-well plates and incubated for 24 h. The cells were stimulated with or without 1 μg/mL of LPS for 30 min and then incubated with 50 μM of nimesulide or the equivalent of nimesulide in HA conjugates for 24 h. After incubation, the culture medium was collected and centrifuged (3000× *g*, 10 min), the supernatant was diluted, and the manufacturer’s instructions were followed.

### 2.14. Statistical Analysis

The quantitative data are presented as the means ± standard deviation. Data were analyzed using Tukey’s or Kruskal–Wallis one-way tests for comparison of the groups, followed by post hoc tests, and a *p*-value of <0.05 was considered as a statistically significant difference.

## 3. Results

### 3.1. Characteristics of HA-Nimesulide Conjugates

**Figure 2:**
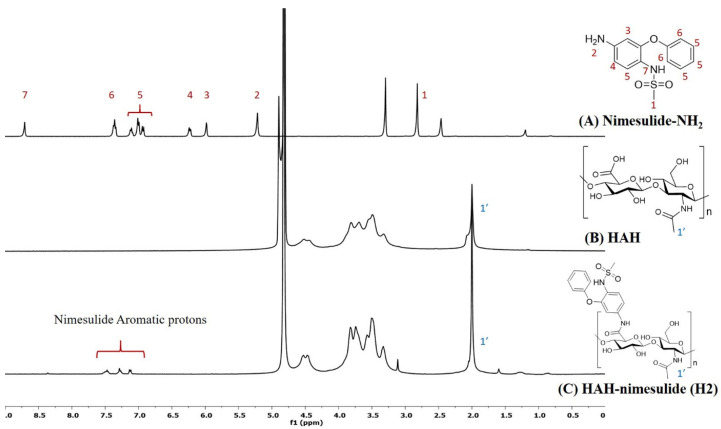
The representative ^1^H NMR spectra of (**A**) nimesulide-NH_2_ (d_6_-DMSO, nt = 200, DMSO: 2.50 ppm), (**B**) HAH (D_2_O, nt = 200, D_2_O: 4.67 ppm), and (**C**) HAH-nimesulide H2 (D_2_O, nt = 200, D_2_O: 4.67 ppm) (Vnmr-400 MHz, Agilent). The molecular structures were shown on the right, and the number assignment of the signals on the NMR spectrum was corresponding to the hydrogen on the structure.

HA-nimesulide conjugates were synthesized from the *N*-[4-amino-2-phenoxyphenyl]methanesulfonamide (nimesulide-NH_2_) with HAH or HAL using a carbodiimide coupling agent, and the structures were characterized by ^1^H NMR spectroscopy. As shown in Figure 2, the characteristic peak of the *N*-acetyl group (–NHCOCH_3_) in HA was identified at 2.0 ppm, and all the aromatic protons of nimesulide in the HA-nimesulide conjugate were identified from a range of 6.8 to 7.6 ppm, consistent with our previous report, thus indicating successful conjugation of nimesulide onto the HA backbone [23]. The degrees of substitution (DS) ratio of nimesulide in HA-nimesulide conjugates was also analyzed by ^1^H NMR spectroscopy in the D_2_O solvent. The DS calculation was determined using the ratio of integral value of methyl group (1′) in the HA monomer and the aromatic protons on nimesulide. The DS ratios of HA-nimesulide conjugates H1, H2, and H3 groups were 4.0%, 4.0%, and 12.5%, respectively (Supplementary Figures S1–S3). The osmolarity of human tears is a marker for diagnosing DES, and the normal range varies between 291 and 316 mOsm/L [28,29], but differences exist between human and animal subjects [30,31,32]. From the experiments, the osmolarity of normal saline, H1, H2, H3, and Optive Fusion^®^ were 296.7 ± 0.5, 303.3 ± 4.2, 299.0 ± 0.8, 318.0 ± 4.5, and 307.7 ± 2.5, respectively (Table 1). The osmolarity of normal saline, H1, H2, and Optive Fusion^®^ was within the range of human tears, and H3 was slightly higher, whereas the osmolarity of H1 and H2 was lower than that of artificial tears.

### 3.2. Dry Eye Diagnosis and Fluorescein Staining on the Ocular Surface

**Figure 3:**
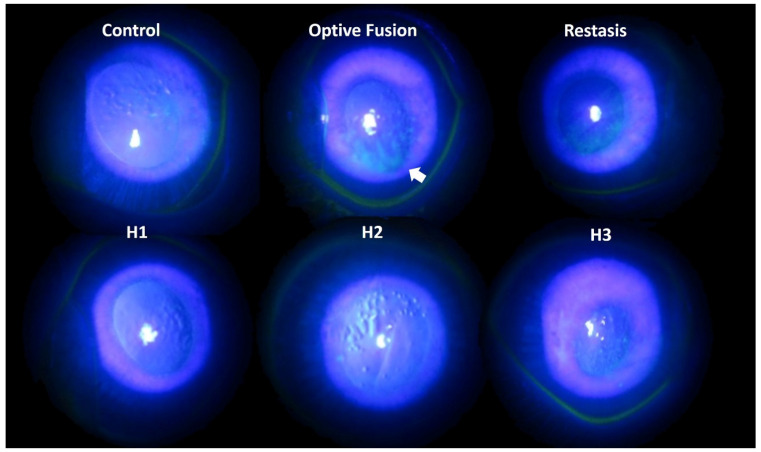
Representative images of corneal fluorescein staining for each group after DES induction with BAC and treatment.

Tear production was assessed by the Schirmer test on days 0, 14, and 28. The mean value of tear production decreased following the administration of BAC solution (data not shown). Fluorescein sodium staining of the ocular surface revealed damaged corneal epithelium by slit-lamp microscopy (Figure 3). In the normal group, almost no fluorescein stain was observed on the ocular surface, which indicated the absence of corneal epithelium damage. By contrast, fluorescein stain was observed on the rabbits’ cornea at day 28 after a four-week application of 0.1% BAC solution. Corneal epithelium damage was characterized by the loss of smooth surface texture and a roughened appearance. A slight fluorescein stain was observed on the ocular surface in the H1, H2, H3, and Restasis^®^ groups after two weeks of treatment. The large plaque of fluorescein stain was still observed in spite of a two-week administration of Optive Fusion^®^. In other words, artificial tears might not create or maintain a suitable environment for the regeneration of corneal epithelium cells in the Optive Fusion^®^ group.

### 3.3. Goblet Cell Density in Conjunctival Impression Cytology

**Figure 4:**
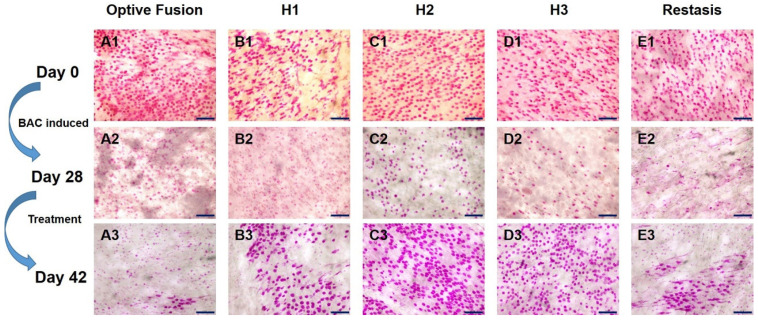
Representative images of PAS-stained conjunctival impression cytology images in BAC-treated eyes for each group on days 0 (**A1**–**E1**), day 28 (**A2**–**E2**), and day 42 (**A3**–**E3**). Goblet cell densities were increased, and the cell shape appeared normal in all treatment groups except for Optive Fusion^®^. Scale bar = 100 μm.

The change in goblet cell density was used to predict the morphological and cytological changes in the conjunctiva [34]. The results from the conjunctival impression cytology showed that the goblet cell density was high and that the goblet cells were oval-shaped before BAC induction (Figure 4A1–E1). After four weeks of BAC induction, the goblet cell density was decreased and was accompanied by a change in shape, which indicated the successful establishment of the dry eye model (Figure 4A2–E2). Except for in the Optive Fusion^®^ group, the goblet cell density was increased, and the cell shape became more oval and fuller in all other treatment groups (Figure 4A3–E3).

**Figure 5:**
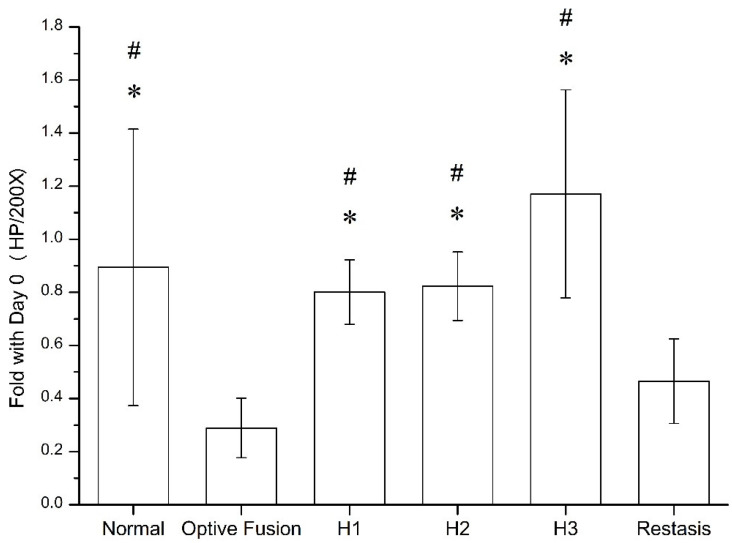
The ratio of goblet cell number in the normal, Optive Fusion^®^, H1, H2, H3, and Restasis^®^ groups on day 42. Significant differences compared to Optive Fusion^®^ (* *p* < 0.05) and Restasis^®^ (^#^ *p* < 0.05) were observed in favor of the HA-nimesulide formulation.

The goblet cell density was calculated by ImageJ software (Figure 5), which was significantly reduced in the Optive Fusion^®^ and Restasis^®^ groups when compared with the normal group; however, the HA conjugate (H1, H2, and H3) groups showed no significant difference compared to the normal group and were significantly higher than that of the Optive Fusion^®^ and the Restasis^®^ groups.

### 3.4. Histology and Immunohistochemistry of Corneal Tissues

**Figure 6:**
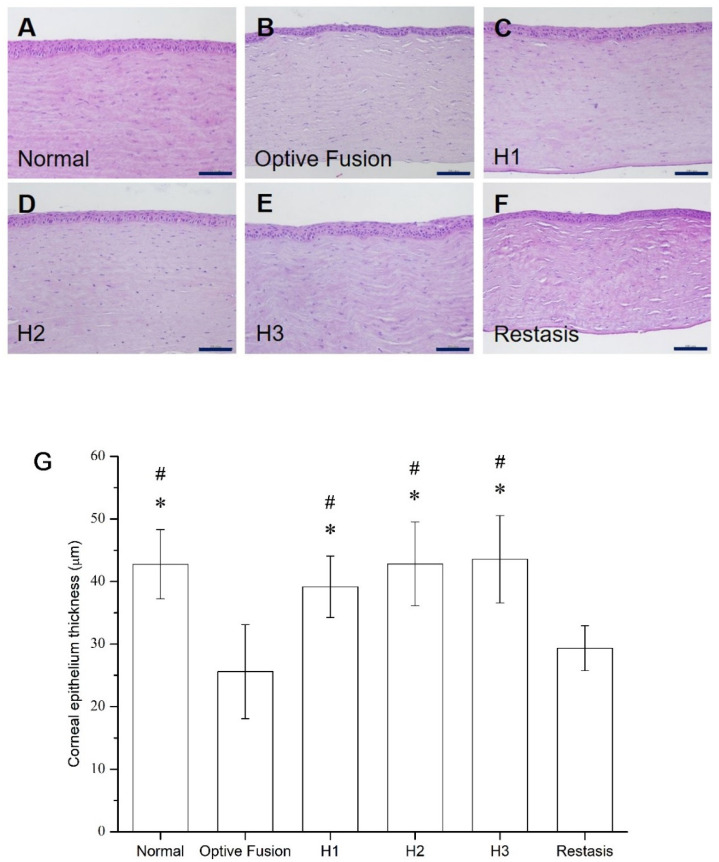
Representative images for histologic examination of the cornea on day 42 in the normal (**A**), Optive Fusion^®^ (**B**), H1 (**C**), H2 (**D**), H3 (**E**), and Restasis^®^ (**F**) groups. The corneal epithelial thickness was preserved in all groups except for the Optive Fusion^®^ and Restasis^®^ groups. (**G**) The average corneal epithelial thickness in each group by ImageJ analysis. The HA-nimesulide groups had preserved thickness, and significant differences were observed when compared with Optive Fusion^®^ (* *p* < 0.05) and Restasis^®^ (^#^ *p* < 0.05). Scale bar = 50 μm.

The average corneal epithelial thickness in dry eye patients has been proven to be thinner than normal people [35,36]. The light microscopy of the corneal sections in the normal group had three to five epithelial layers (Figure 6A), and the corneal epithelial thickness was comparable to the normal group in all HA-nimesulide treatment groups (Figure 6C–E); in contrast, the epithelial thickness was reduced in the Optive Fusion^®^ and Restasis^®^ groups (Figure 6B,F). The average thickness of the epithelial layer in all groups was measured by ImageJ software, and the results are shown in Figure 6G. The HA conjugate (H1, H2, and H3) groups showed no difference from the normal group, the Optive Fusion^®^ group was significantly thinner in comparison to the normal, H1, H2, and H3 groups, and similar results were observed for the Restasis^®^ group.

**Figure 7:**
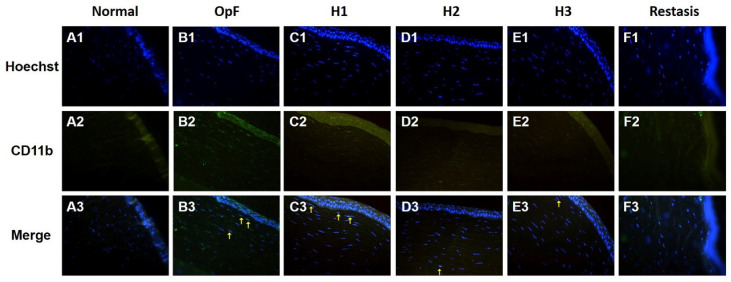
Representative images of immunofluorescence staining of corneal sections on day 42 showing CD11b staining (green, **A2**–**F2**), with Hoechst 33342 nuclear counterstaining (blue, **A1**–**F1**) and merged image (**A3**–**F3**) as an indicator of corneal inflammation in the normal (**A**), Optive Fusion^®^ (**B**), H1 (**C**), H2 (**D**), H3 (**E**), and Restasis^®^ (**F**) groups. HA-nimesulide conjugates exhibited a similar anti-inflammatory effect as Restasis^®^.

The infiltration of inflammatory cells in the cornea was evaluated by CD11b immunofluorescence staining, which is a macrophage marker. As shown in Figure 7, no macrophages infiltrated the cornea in the normal group, whereas CD11b-positive cells were present in the corneal stroma of the Optive Fusion^®^ group. Although the green fluorescence of CD11b appeared in the H1, H2, H3, and Restasis^®^ groups, the administration of HA-nimesulide conjugates and Restasis^®^ markedly reduced the extent of infiltration by CD11b-positive cells, which indicated that the HA-nimesulide conjugates might have a similar anti-inflammatory effect as Restasis^®^.

### 3.5. In-Vitro Evaluation of HA-Nimesulide Conjugates in Raw 264.7 Cell Line

**Figure 8:**
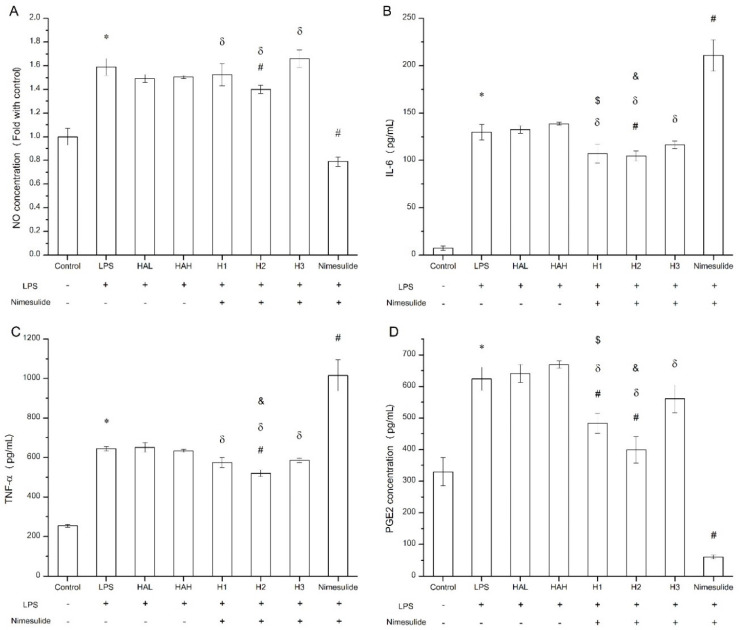
Anti-inflammatory evaluation of free HA, nimesulide, and HA-nimesulide conjugates in LPS-stimulated Raw 264.7 cells displayed as (**A**) the ratio of nitric oxide concentration compared with control group, and (**B**) IL-6, (**C**) tumor necrosis factor alpha, and (**D**) PGE2 concentrations in the culture medium. * *p* < 0.05 versus the control group. ^#^ *p* < 0.05 versus the LPS group. ^*δ*^ *p* < 0.05 versus the nimesulide group. ^*$*^ *p* < 0.05 versus the HAL group. ^*&*^ *p* < 0.05 versus the HAH group.

The secretion of pro-inflammatory cytokines and mediators such as nitric oxide and PGE2 by macrophages following LPS challenge can be used to evaluate the anti-inflammation effect in vitro [37,38]. The viability of Raw 264.7 cells exposed to free HA, nimesulide, and HA-nimesulide conjugates at various concentrations (400, 200, 100, 50, and 25 μM) is shown in Supplementary Figure S4, where 50 μM of nimesulide or equivalent in the HA-nimesulide conjugates resulted in more than 80% viability, and thus this concentration was selected for subsequent assays. Figure 8 shows the anti-inflammatory effect of free HA, nimesulide, or HA-nimesulide (H1, H2, H3) in Raw 264.7 cells. Firstly, LPS successfully stimulated nitric oxide, IL-6, TNF-α, and PEG2 production compared with those in the control group (* p < 0.05). Secondly, both free nimesulide and H2 conjugates significantly reduced nitric oxide production following LPS challenge, whereas no significant responses were observed in the free HA, H1, and H3 (*^#^ p* < 0.05). In particular, free nimesulide led to a significant decrease in nitric oxide level compared with H1, H2, and H3 of the HA-nimesulide conjugate-treated groups (*^δ^ p* < 0.05) (Figure 8A). Thirdly, H2 conjugates significantly reduced IL-6 and TNF-α secretion, whereas free nimesulide apparently increased amounts of both cytokines compared with those in LPS-stimulated groups. Intriguingly, the H1 and H2 groups showed a significant inhibition in IL-6 secretion compared to the HAL (HA with 36 kDa) (*^$^ p* < 0.05) or HAH (HA with 360 kDa) groups (*^&^ p* < 0.05) (Figure 8B,C). Finally, free nimesulide, H1, and H2 HA-nimesulide conjugates significantly inhibited the production of PGE2 compared with that in the LPS-stimulated group (*^#^ p* < 0.05), however, the inhibitory activity of H1 and H2 was lower than that of free nimesulide (*^δ^ p* < 0.05). Thus, taken together, these results indicate that HA-nimesulide conjugates exhibit an anti-inflammatory activity, especially in the H1 and H2 groups, and we speculated that the anti-inflammatory mechanisms of HA-nimesulide might differ from those of free nimesulide.

## 4. Discussion

Currently, artificial tears are considered the first-line therapy for dry eye, which serve to alleviate symptoms of dry eye discomfort by hydrating the cornea with water and components with high water-retention properties, such as methylcellulose and HA. However, several factors, such as inflammation, reduced tears, and excessive evaporation of tears, might result in inconsistent responses to artificial tears in clinical tests [18,19,39,40,41]. In recent years, a number of studies have been conducted on various animal models of dry eye, such as simulating excessive tear evaporation by reducing environmental humidity [42], administering antimuscarinic agents such as scopolamine or atropine to reduce the amount of tear secretion [14,43,44,45], or BAC to cause damage and apoptosis of ocular cells, which in turn creates an inflammatory environment [46,47,48,49,50]. The mechanism of artificial tear treatment was inconsistent from the inflammatory animal model, which might lead to a reduction in the therapeutic effect; moreover, similar results were reported using the BAC-induced experimental model [21,51].

Restasis^®^ is currently the second-line drug for DES because pro-inflammatory factors and infiltration of inflammatory cells were found on the ocular surface and in tear fluids in dry eye disease, and local immunosuppression by Restasis^®^ prevented the production of inflammatory factors, such as interleukin-2 (IL-2). The subsequent reduction in IL-2 levels also reduced the function of effector T cells. Additionally, the administration of Restasis^®^ reduced the ocular surface disease index in clinical trials [52,53,54,55]. Our results suggested that Restasis^®^ was superior for reducing CD11b-positive cells in an experimental dry eye model (Figure 7). In addition, according to a previous clinical report, the goblet cell density was significantly greater than that of the baseline in the inferior bulbar conjunctiva after a 6-week treatment of cyclosporine emulsion [41], however, this treatment effect, like those reported elsewhere, was not observed in our study using the BAC induction model. The difference might result from the relatively short-term exposure to an agent with a higher preservative concentration than that found in commercially available eye drops [56].

According to a previous report, the level of PGE2 in the tears of dry eye patients was higher than normal, and this compound plays an important role in inflammation [57], which suggests the potential for treatment with the anti-inflammatory drug nimesulide. Although nimesulide could inhibit COX-2 expression and the biosynthesis of PGE2 when applied, the low solubility of nimesulide in water necessitates the addition of DMSO to the ophthalmic agent, despite its possible adverse effects on dry eye [58]. However, whether or not DMSO has adverse effects on the eye in the ophthalmic agents remains inconclusive [59,60]. In our study, the HA-nimesulide conjugate system regulated the drug concentration and improved the solubility in normal saline without any organic solvent.

Common adverse events reported in several clinical studies using topical cyclosporine include ocular pain (11.0%) and burning and irritation (13.6%), and as many as 26.67% of patients withdrew from treatment as a result of intolerable side effects [7,61,62]. Restasis^®^ consists of a complex emulsion, and the drug and other components can distribute themselves in different phases based on their properties, such as the water phase, micellar phase, microemulsion phase, or oil/water interface [63]. It is reported that no tolerability issues occurred with increasing concentrations of cyclosporine in ocular tissue, and thus the issue of side effects and patient tolerability with Restasis^®^ might be related to the composition of the formulation instead of the drug itself [8]. In contrast to the complex systems, we developed the HA-nimesulide conjugate system by covalently attaching small-molecule drugs to HA without additional surfactants. Thus, we could avoid patient compliance and tolerance issues stemming from additives.

It has been previously demonstrated that inducible nitric oxide synthase and COX-2 can be induced by LPS, thus, increased nitric oxide and PGE2 levels in macrophages, and the expressions of pro-inflammatory cytokines such as IL-6 and TNF-α, are associated with NF-κB activation and thus inflammation [38,64]. As a COX-2 inhibitor, nimesulide efficiently inhibited PGE2 and nitric oxide production in LPS-treated Raw 264.7 cells (Figure 8A,D), which was consistent with a previous report [65]. For the modulation of IL-6, we found that nimesulide significantly increased IL-6 secretion in LPS-treated Raw 264.7 cells (Figure 8B), and this phenomenon was demonstrated by Ramalho et al. [66], whereas downregulation of IL-6 concentration by nimesulide was also reported [67]. Recently, Gungot et al. demonstrated that nimesulide derivatives with various sulfonamide or amide moieties had different COX-2 inhibition abilities [68]. For HA-nimesulide conjugation, the amide covalent bond between the polysaccharide and nimesulide may alter the inhibitory activities of free nimesulide. Thus, it was found that although HA-nimesulide conjugates, such as H2, significantly inhibited the levels of PGE2 and nitric oxide, they were not as efficient as free nimesulide (Figure 8A,D). Interestingly, HA-nimesulide conjugates revealed different activities than free nimesulide in the inhibition of pro-inflammatory cytokines (Figure 8B,C), indicating the possibility of different mechanisms in the HA-nimesulide groups. Moreover, it is reported that the degradation of HA by hyaluronidases can give rise to approximately 20 kDa cleaved-products, such as disaccharide units and smaller oligosaccharides, and the degraded HA-drug products might improve the pharmacological activities [69,70]. Therefore, we speculated that the mechanisms of action of HA-nimesulide may be different from those of nimesulide. Detailed investigations of anti-inflammatory activities, pharmacological activities of HA-nimesulide conjugates, and their metabolites are ongoing in our group.

In ophthalmology, it has been reported that HA with a low molecular weight improves the ability of macrophages and dendritic cells to produce proinflammatory cytokines. Conversely, HA with a high molecular weight exhibited more benefits in preventing corneal epithelium damage and modulating macrophage activity upon binding to the CD44 receptor [71,72,73,74,75]. The expression of the CD44 receptor increases when corneal epithelial cells are damaged, and the CD44 protein enhances wound-healing by regulating the regeneration and migration of epithelial cells after binding with HA [76]. Moreover, HA with a high molecular weight prolongs the drug-retention time on the corneal surface due to its mucoadhesive ability and viscosity [77]. Our results demonstrated that the H2 group with 360 kDa molecular weight HA promoted the recovery of conjunctival goblet cells more than the H1 group with 36 kDa molecular weight HA. Based on staining, the H2 group decreased the CD11b-positive cells more than the H1 group, despite both having the same degree of substitution (DS) and thus the equivalent amount of nimesulide. Besides, the results in the H2 group were similar to those of the H3 group, even if the dosage of nimesulide in the H3 group was 30-fold higher. In general, we speculate that the 360 kDa molecular weight HA may provide a longer drug-retention time, which may result in sustained release of the therapeutic agent on the eye, and this HA conjugation system may potentially be used for the controlled release of topical ocular medications.

## 5. Conclusions

In this study, we successfully facilitated the introduction of a hydrophobic NSAID, nimesulide, as eye drops using the hyaluronic acid conjugation technique. The HA-nimesulide conjugates significantly increased the solubility of nimesulide in normal saline without the need for an additional organic solvent, and enabled the potential introduction of nimesulide as aqueous eye drops. The HA-nimesulide conjugates improved the density of goblet cells and the recovery of average corneal epithelial thickness when compared with commercial products Optive Fusion^®^ and Restasis^®^ in a BAC-induced experimental dry eye rabbit model, and reduced the infiltration of CD11b-positive cells comparable to the immunomodulator Restasis^®^. In addition, the production of pro-inflammatory cytokines and the PGE2 metabolite was reduced. However, the mechanism of action of HA-nimesulide conjugates is unclear and may be different from nimesulide. In conclusion, we demonstrated the potential of the HA-nimesulide conjugate as topical anti-inflammatory eye drops for the application in DES.

## Figures and Tables

**Table 1:** The osmolarity of human tears, artificial tears, and HA conjugate solutions.

	Osmotic Pressure (mOsm/kg)
Human tears	308.0 ± 12.0 [33]
Normal saline	296.7 ± 0.5
H1	303.3 ± 4.2
H2	299.0 ± 0.8
H3	318.0 ± 4.5
Optive Fusion^®^	307.7 ± 2.5

## Data Availability

The data presented in this study are available on request from the corresponding author.

## Supplementary Materials

The following are available online at https://www.mdpi.com/article/10.3390/pharmaceutics13091366/s1, Figure S1: ^1^H NMR spectra of 36 kDa HA-nimesulide with 4% grafting ratio, Figure S2: ^1^H NMR spectra of 360 kDa HA-nimesulide with 4% grafting ratio, Figure S3: ^1^H NMR spectra of 36 kDa HA-nimesulide with 12% grafting ratio, Figure S4: The cell viability of HA-nimesulide conjugates series in the Raw 264.7 cell line for 24 h.
