# Supplementary material for: Effects of Eye Drops Containing Hyaluronic Acid-Nimesulide Conjugates in a Benzalkonium Chloride-Induced Experimental Dry Eye Rabbit Model
Source: Pharmaceutics. 2021 Aug 30;13(9):1366. doi: 10.3390/pharmaceutics13091366 (PMC8469214; doi:10.3390/pharmaceutics13091366)
Supplement: Supplementary file 1 [file pharmaceutics-13-01366-s001.zip › pharmaceutics-1330747-supplementary.pdf]

pharmaceutics

Supporting Information: Effects of Eyedrops Containing Hyaluronic Acid-Nimesulide Conjugates in a Benzalkonium Chloride-Induced Dry Eye Rabbit Model

Tzu-Yang Chen, Ching-Li Tseng, Chih-An Lin, Hua-Yang Lin, Parthiban Venkatesan and Ping-Shan Lai

The degrees of substitution (DS) ratio of nimesulide in hyaluronic acid (HA)-nimesulide conjugates were analyzed by ^{1}H-NMR spectroscopy (Vnmr-400 MHz, Agilent) studied with D_2O as a solvent. (nt = 200, lb = 1, D_2O: 4.67 ppm). The DS calculation was using the following methods. The integral value of -CH_3 in the HA monomer and -H in the drug's benzene ring were used to compute the DS ratio. The integral value of -CH_3 in HA monomer was normalized as 300.00, and the DS ratio of HA-nimesulide conjugates was defined as the sum of integral value in benzene ring divided by the number of hydrogens on the benzene ring, in other words, the number of nimesulide per HA disaccharide unit. The ^{1}H NMR spectrum of H1, H2, and H3 was shown in Figure S1, Figure S2, and Figure S3, and the DS ratio was 4%, 4%, and 12% respectively.

Initially, the effect of free nimesulide, free HA, and HA-nimesulide conjugates (H1, H2, H3) on viability of Raw 264.7 cells was determined using an 3-(4,5-dimethylthiazolyl)-2-2,5-diphenyltetrazolium bromide (MTT) reagent. The cells at a density of 10^4 cells/well were seeded in 96-well plates. After the 24-hr incubation, indicated concentrations of nimesulide or equivalent of nimesulide ranged from 400 μM to 25 μM, and HA ranged from 3.8 mg/mL to 0.3 mg/mL were added to the cells and incubated for an additional 24-hr. The MTT solution was added to each well and incubated for 4-hr at 37°C. After removing the MTT solution, DMSO was added to each well to dissolve the formazan. The absorbance was measured using a scanning multiwell plate reader at 570 nm (SpectraMax® M2e, Molecular Devices LLC, USA). The cell viability examination was demonstrated in Figure S4. The result revealed more than 80% survival rate of all testing groups up to 50 μM, thus, this specific concentration was selected for subsequent assays.

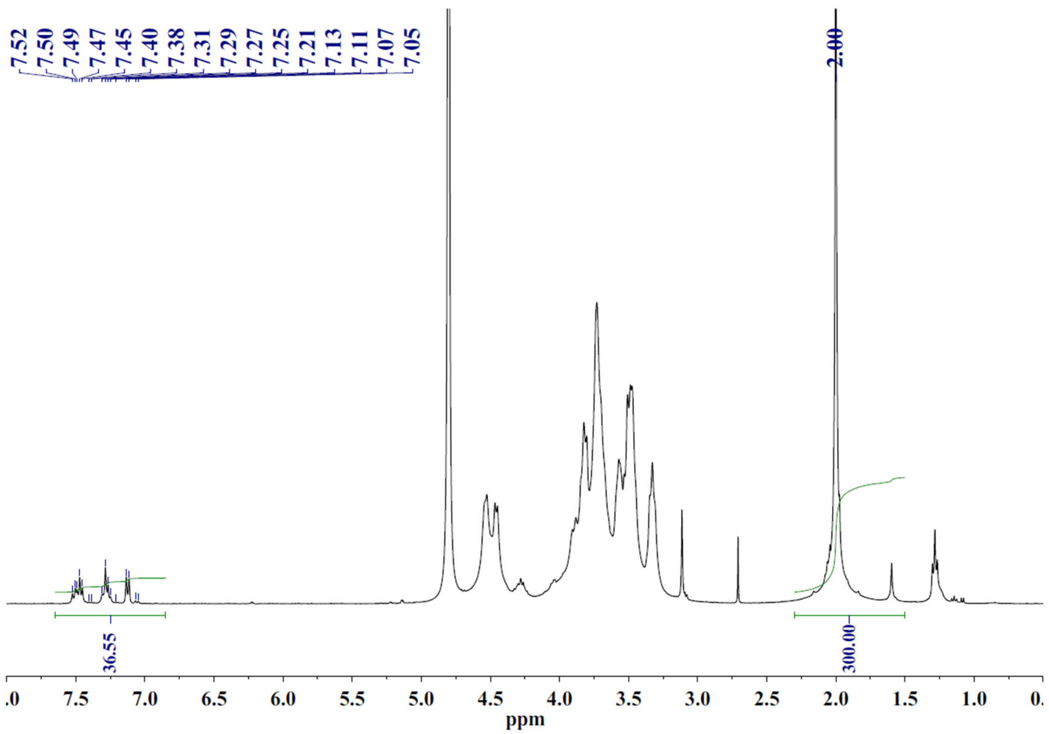

Figure S1. ^{1}H NMR spectra of 36 kDa HA-nimesulide with 4% grafting ratio (H1). (nt = 200, D_2O: 4.67 ppm).

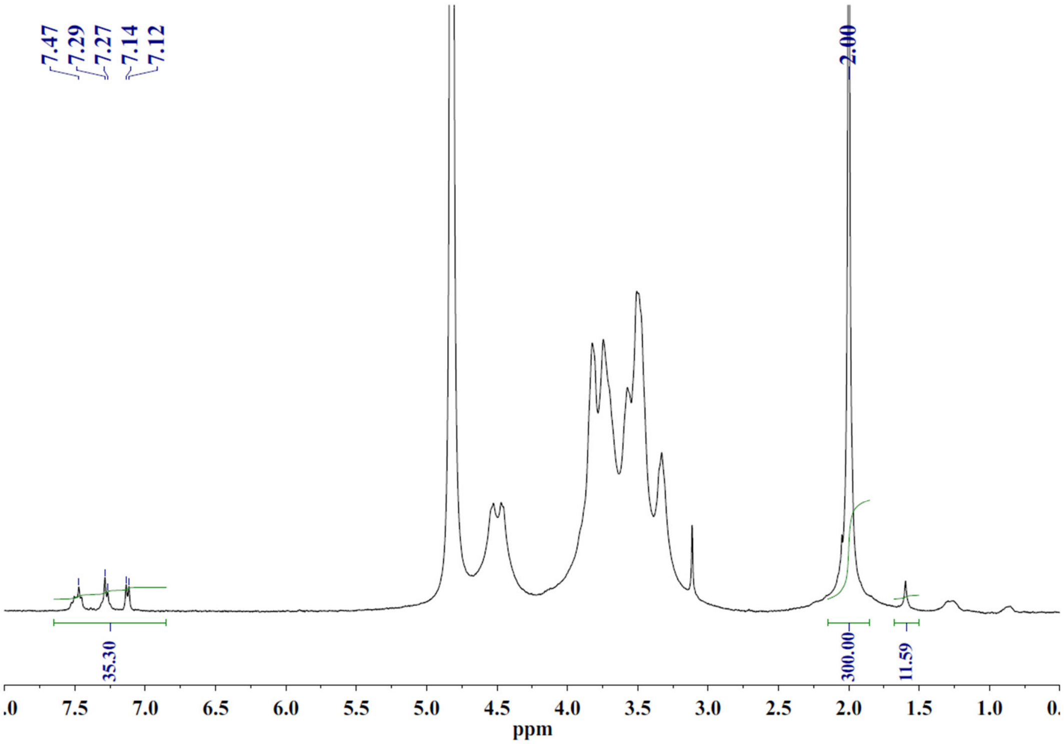

Figure S2. ^{1}H NMR spectra of 360 kDa HA-nimesulide with 4% grafting ratio (H2). (nt = 200, D_2O: 4.67 ppm).

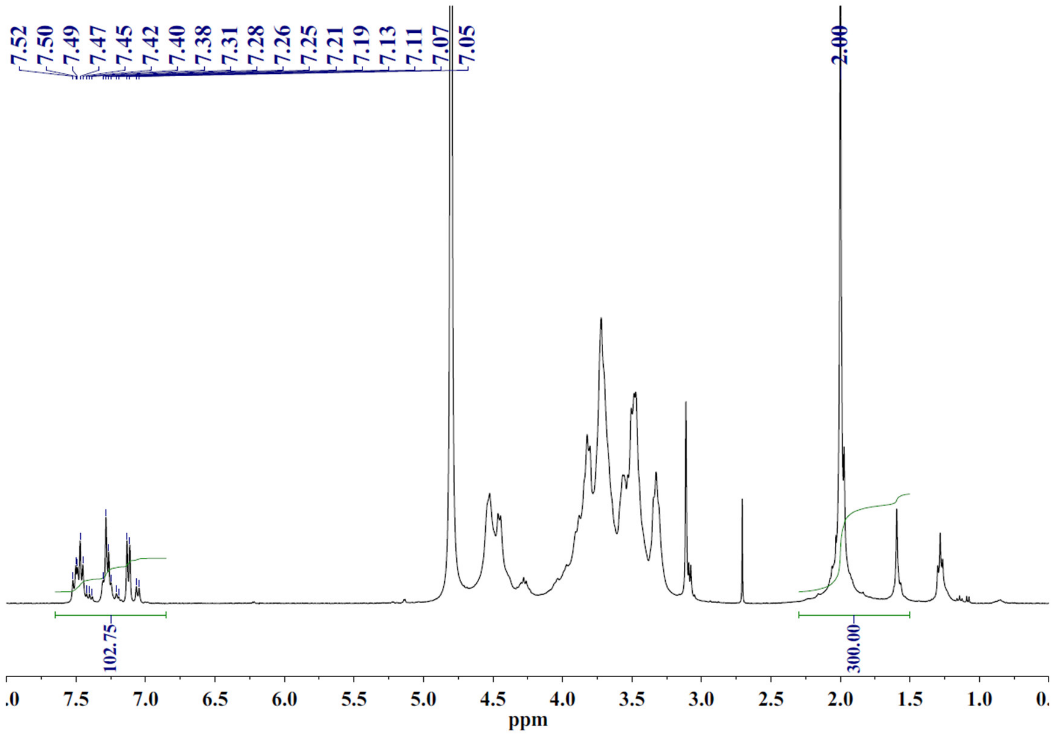

Figure S3. ^{1}H NMR spectra of 36 kDa HA-nimesulide with 12% grafting ratio (H3). (nt = 200, D_2O: 4.67 ppm).

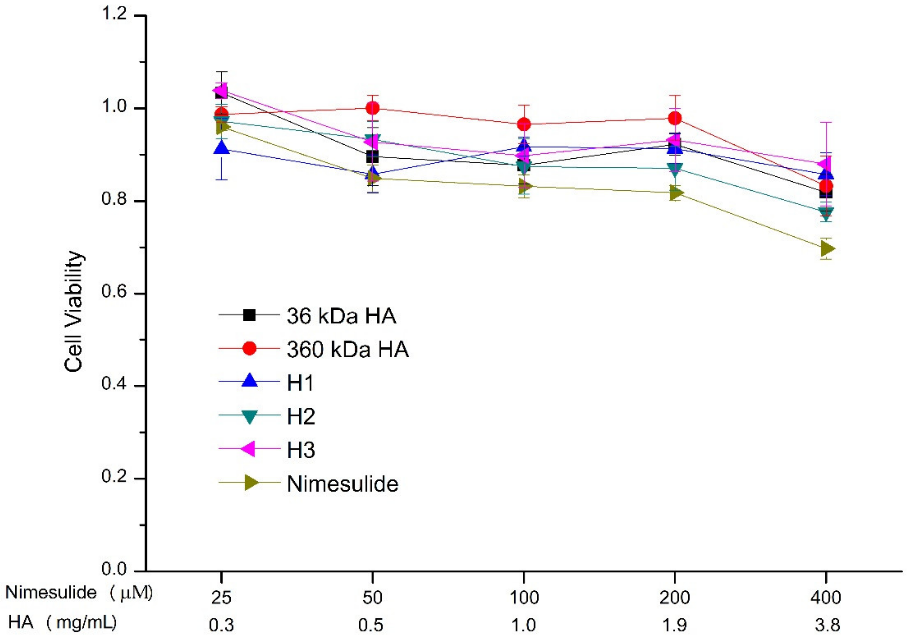

Figure S4. The cell viability of HA-nimesulide conjugates series in Raw 264.7 cell line for 24-hour.
